# On the Structure of the Inductive Syllogism and Its Correlation to the Deductive

**Published:** 1861-04

**Authors:** R. G. Latham


### Art. VIII.—ON THE STRUCTURE OF THE INDUCTIVE SYLLOGISM AND ITS CORRELATION TO THE DEDUCTIVE.

By R. G. Latham, M.D., F.R.S., &c.

In the present paper the structure of the Inductive Syllogism will be investigated, the details of which will be considered from two points of view. They will, of course, be treated as what they are in themselves; but this will scarcely form the main part of the inquiry. The main part of the inquiry, if it were not for an extract which will soon be given, would lie in the relation they bear to the syllogism of the ordinary moods and figures; this relation being a *cor*relation. As it is, however, it will consist of something else.

Let two series of facts not only give a correlation, but let that correlation be distinctly recognised by the first systematic expositors of the science upon which they bear, and the result will be a certain amount of harmony and symmetry in the terms which such expositors either adopt or invent. This is because the correlation itself is one of the phenomena to which they will have to attend. Let the correlation, however, be overlooked, and the language will be adapted to the subject-matter under notice alone; its relations to anything else being ignored. This has been the case with the ordinary syllogism. Its terms were framed with a view to itself only.

Now the main object of the present paper is to correct this exclusiveness, and to modify the language of the text-books to the extent required for the full exhibition of the correlation in question.

The Deductive Syllogism begins with a proposition like

*All men are mortal,*

and having, *secundum artem,* added the word *Socrates,* concludes with

*Socrates is mortal,*

descending from the larger, to the smaller, class.

The Inductive Syllogism, on the other hand, begins with

*Socrates is mortal;*

and having, *secundum artem,* added *men,* concludes with

*All men are mortal.*

In this we begin with the smaller, and end with the larger, class. If so, the extremes are the same, not only in their elements, but in the arrangement of them, though they differ widely in the place that they take in the syllogism. The major premiss of the one is the conclusion of the other, and *vice versâ.* How, then, is this brought about? That depends upon the form of the intermediate proposition; which is, in the first case, *Socrates is comprehended in the class man:* in the second, *the whole class man, in respect to its mortality, is adequately represented by Socrates.*

All this is neither more nor less than what has been better said by Sir William Hamilton already; the following extract—the one alluded to above—being from his article Logic in reference to the recent English Treatises on that Science. (*Edinburgh Review,* April, 1833). Reprinted (1852) in his *Discussions on Philosophy,* &c.; see pp. 156—165.

"Not only is the Deductive thus, in a general way, dependent for its possibility on the Inductive Syllogism; the former is, what has not been observed, in principle and detail; in whole and in part—in end and in means—in perfection and in imperfection, precisely a counterpart or inversion of the latter.

"The Inductive inference is equally independent, and though far less complex, equally as worthy of analysis as the Deductive. It is governed by its own laws, and if judged aright, must be estimated by its own standard. The correlation of the two processes is best exemplified by employing the same symbols in our ascent through an Inductive, and our re-descent through a Deductive Syllogism.

*Inductive.*

X, Y, Z, are A.
X, Y, Z, are (whole) B.
Therefore B is A.

*Deductive.*

B is A.
X, Y, Z, are (under) B.
Therefore X, Y, Z, are A.

or—

A contains X, Y, Z.
X, Y, Z constitute B.
Therefore, A contains B.

or—

A contains B.
B contains X, Y, Z.
Therefore A contains X, Y, Z.

These two syllogisms exhibit, each in its kind, the one natural and perfect figure. This will be at once admitted of the Deductive, which is in the first figure. But the Inductive, estimated, as it has always been, by the standard of the Deductive, will appear a monster. It appears, on that standard, only in the third figure, and then, contrary to the rule of that figure, it has an universal conclusion. But when we look less partially and more profoundly into the matter, our conclusion will be very different.

"In the *first* place, we find that the two syllogisms present so systematic a relation of contrast and similarity, that the perfection of the one being admitted, we are analogically led to presume the perfection of the other.

"In the *propositions*, the order of the terms remains unchanged; but the order of the propositions themselves is reversed; the conclusion of the one syllogism forming the major premiss of the other.

"Of the *terms*, the major is common to both; but (as noticed by Aristotle), the middle term of the one is the minor of the other. In the common minor premiss, the terms, though identical, have, with the different nature of the process, changed their relation in thought. In the Inductive, the parts being conceived as constituting the whole, are the determining notion; whereas, in the Deductive, the parts being conceived as contained under the whole, are the determined."

That this (*inter alia*) gives the correlation in question, with full and proper clearness, is evident; and it may also be added, that the remarks by which it is preceded give the difference between the Logic of the Inductive Syllogism (the special object of the present paper), and Induction in the ordinary, but somewhat catachrestic, sense of the term, in a manner which few would attempt to improve upon.

Let us address ourselves, then, to what stands over—viz., the question as to how far the present language, which was framed to suit the exigencies of the Deductive Syllogism alone, requires modification when the Inductive Syllogism, with its correlation, comes within the field of our inquiry. Let us get such harmony and symmetry as can be obtained at a moderate amount of innovation.

At present we have got a transposition, and something else. We have also got three subjects *without* signs; and we have also got the statement that the Inductive Syllogism is "far less complex" than the Deductive. But we have no exposition of the grounds upon which this difference of complexity rests, nor (what is more important) any evidence to show that it exists at all. In fact, it does *not* exist. So far as the ordinary syllogism is deductive, each and all of its complexities have their counterparts in the inductive. It is only, however, *in part* that the ordinary syllogism is either deductive or inductive at all; so that the identification of it, in its common form, with deduction —to the exclusion of aught else—is exceptionable; and equally exceptionable is the comparison of the few syllogisms which are truly inductive, with syllogisms in general. These last are partly deductive and partly something else; the deductive portion alone being that by the side of which the inductive should be placed by any one who would measure either the relative importance or the relative complexity of the two.

Let the ascent from the less to the more, and the descent from the more to the less, general be the essential characteristics of Inductive and Deductive inference, and let—

*All men are mortal;*
*All men are rational;*
*Some rational beings are mortal;*

be tested by this criterion. Where is the ascent and descent? Where is the more and less particular? Where the greater generality? Where, in short, is the subalternation of classes which in

*All men are mortal;*
*All heroes are men;*
*All heroes are mortal;*

gives us *mortal* as the name of a class more general than that of *men,* and *men* as the name of a class more general than of *heroes;* classes of which the greater, or major, contains the middle, whilst the middle, in *its* turn, contains the smaller, or minor. Here all the classes have a definite relation, as either great or less, to each other; and ascent or descent (one or both) can be effected throughout.

In—

*All men are mortal;*
*All men are rational;*
*Some rational beings are mortal;*

between two of the classes—the one expressed by *mortal,* and the one expressed by *rational*—there is no comparison of generality at all. For all that the syllogism tells us, *mortal* may be more general than *rational,* or *rational* more general than *mortal.* There is no way of measuring the two. Instead of one of them including the other as a class, they simply meet in a third. Each may be of any magnitude; the only thing concerning them that we know, being this—they *may* be equal. They may be equal, inasmuch as there is no proven inequality. Of the class denoted by *man*, we know a little more. As it is the subject in each premiss, it cannot be greater, and may be less than its predicate.

If all this be true, the syllogisms of the third figure are *other than deductive;* so are those of the second. In—

*No bad man is happy;*
*Some tyrants are happy;*
*Some tyrants are not bad men;*

the two subjects of the premisses stand in the same relation to one another as stood the two predicates of the preceding syllogism. The class expressed by *tyrant* may be larger than that expressed by *bad men*, or it may be smaller; inasmuch as the syllogism never measures the one against the other. In practice it treats them as equal; no inequality being proven, or even suggested. Meanwhile of *happy*, as the name of a class, we know, *mutatis mutandis*, what we know of *men*. Mark the words *mutatis mutandis*. As the predicate in each premiss, it cannot be less, and may be greater, than its subject.

Like those, then, of the third, the syllogisms of the second figure are *other than deductive*.

With these limitations—limitations by which the deductive portion of the ordinary syllogism is restricted to the first figure—the difference in the amount of detail between the systems disappears. Induction is on a level with Deduction; Deduction being smaller than it is supposed to be.

Meanwhile the question of the *signs*, in which is involved that of the copula, calls for notice. That there are no signs to the predicates of the syllogisms of the extract has already been stated.

The ordinary copula is *is*, or *is not; are*, or *are not*. If, however, the substitution for it of such words as *contain*, *constitute*, *are under*, and *are* (*whole*), is not thought to give too great a departure from the common form by a logician like Sir William Hamilton, it is not likely to be objected to by the world at large. The present writer would, perhaps, prefer *represent* (or *stand for*) to *constitute* and *are* (*whole*); whilst, instead of *are under*, he would write *stand as*. He would also connect with it, *as far as* n *or* m *is concerned*—*n* or *m* being the predicate of the other premiss—whatever it may have been.

For the forthcoming observations Sir W. Hamilton's copulas may stand as they are; and upon these it must be remarked that *constitute* and *are whole* are not in the same category. They are not equally copular; or, if they are, they fail to strike us as such. Each contains a predicate element, but this predicate element is far more conspicuous (or less latent) in the one than the other. In *x, y, z are* (*whole*) *B*, we have something very like *x, y, z are all-B;* where *are* is a simple copula, and *all-B* a quantified predicate. That *x, y, z constitute B*, is really *x, y, z are that which constitutes B*, is true. But, as there is nothing which exhibits the sign *all*, the predicative element in this case obtrudes itself less.

Let the copular element of $x, y, z = B$ predominate, and the inversion that changes

*All men are mortal;*
*Socrates is a man;*
*Socrates is mortal;*

is—

*Socrates is mortal;*
*All men are as Socrates;*
*All men are mortal;*

the alteration consisting in a transposition of the extreme terms along with one of the predicate and subject of the middle one; the *sign* remaining as before—*i.e.*, attached to the subject.

In this case the original Figure is preserved, and both the Deductive and the Inductive Syllogisms are in the First.

Not so, however, if the predicate element assert itself. When it does this,

*Socrates is mortal;*
*Socrates stands for all men;*
*All men are mortal;*

is the result. Here the syllogism is in the third figure, and a predicate is quantified. This means that the subject and the predicate of the middle proposition have been left as they were, the *sign* alone having changed its place.

In this way we get an Inductive Syllogism of the First Figure, and another in the Third; the first corresponding (*nearly*) with *Barbara*, the second (*nearly*) with *Datisi*.

Why do I say nearly? Let the typical form of the syllogism, whether Deductive or Inductive, be that wherein there is the *maximum* of ascent and descent—ascent from the less to the more, descent from the more to the less, general. In its typical form the Deductive Syllogism begins with a Universal, and ends with an Individual, affirmation. In its typical form the Inductive Syllogism begins with an Individual and ends with a Universal. *All men are mortal, and Socrates, being a member of the class man, is mortal,* says Deduction. *Socrates is mortal, and, as Socrates, in the matter of mortality, adequately represents mankind at large, all men are mortal,* says Induction. Each case, however, is extreme. What we say of the class *men* we say of the whole of it: whilst *Socrates* is the name not only for a part of that class, but for the smallest part into which it can be divided.

The more general the one extreme, and the more particular the other, the more typical the syllogism; the most particular sort of particularity being that which is given by an individual. Replace *all* by *some*, and the Universality of our more general extreme is only approached approximatively. Instead of *Socrates*, write *Some philosophers*, and you have only an approach to the *maximum* of particularity.

But what if the typical syllogism as it has just been exhibited be typical only in the eyes of the unlearned? That Socrates is an individual their common sense tells them; which also tells them that an individual is the smallest, the most partial, or the most *particular*, part of the class to which he belongs. And their common sense is right. If *some philosopher* be a particular statement, *one* philosopher is more particular still. It is the part of a part. This is the view of the reader who knows nothing of Logic.

With the slightest tincture of Logic, however, he will think differently. He will know that, in the eyes of the logician, such a statement as *Socrates is a man* is treated, not as a particular proposition, but as a universal one. This is because *Socrates* means *All Socrates.*

It is not difficult to see how these two views may be reconciled. If we look upon an individual object with reference only to itself, it is a *whole.* If we look upon it as a member of a class, it is a *part.* A part of Socrates is an arm, a leg, or what not? something different in kind from Socrates himself. The parts of the class to which Socrates belongs are philosophers, Greeks, human beings, or the like, one of which is Socrates.

Socrates, then, is a whole or a part, according to the view that is taken of him; and, according as this view changes, he is, in the eyes of the logician, in such a proposition as

*Socrates is a man,*

a Universal subject or a Particular one.

Now it is absolutely necessary for the Inductive Syllogism that its *individuals* should be *particular and not universal:* in other words, its individuals must be looked upon with respect to their relations to the class which they represent, rather than with respect to what they are as wholes to their own constituent parts. We cannot, in Induction, make *Socrates* less particular, (*i.e.*, more universal) than *some philosophers.*

Here, then, is a point where a discord has to be got over. How? I draw attention to the fact that the ordinary practice of prefixing to names other than individual (abstract names and certain approximations to them being considered as such) a sign in *both* premisses is unnecessary. For a particular affirmative, the only conditions are that *both* the premisses should be affirmative, and that *one* should be universal. In a syllogism with a universal conclusion *some* never enters at all. Meanwhile, *Darapti*, with its two universals, gives us neither more nor less than what is given us by *Disamis* and *Datisi* with only one. For this reason, *a sign for a particular conclusion is needed in one premiss only*, whilst it is wholly excluded from a universal one. This means that it is never needed in a second premiss at all, except, of course, so far as it may be required for the purposes of language. With or without it, the conclusion is the same. Such being the case, we are free to adapt our signs to the expression of this individuality—individuality rather than simple particularity—which characterizes the Inductive Syllogism.

When the connexion is copular rather than predicative, and the syllogism is in the first figure, no change of sign is required. When, however, it is predicative rather than copular—*i. e.*, when the predicate is quantified and the syllogism is in the third figure—we must individualize it. When the term is a proper name, it is already individual; and to this character of a proper name, or individual term, it must be approximated when it conveys the notion of either more objects than one, or a single object of which the identity is doubtful. Indeed, strictly speaking, such expressions as *Socrates is man*, *Socrates is mortal*, are not absolutely unexceptionable. How do we know that the *same* Socrates is meant?

Let the word *same* be used as a sign in one premiss, and (although it is not necessary to do so) let us say *certain* rather than *some* in the other. This is because in induction we have not only a definite number of objects which form the basis of our reasoning, but have them in the mind at once. When we argue from the individual we argue from *this* or *these*; *that* or *those*; *this*, *that* and *the other*; and our syllogism is—

*This, that, and the other, are mortal;*
*The same stand for all men;*
*All men are mortal.*

Or we may say—

*Certain individuals are mortal;*
*The same stand for all men;*
*All men are mortal.*

In this use of the word *same* there is nothing which good logicians have not allowed, as may be seen in Lambert's *Dianoiologie* amongst the older, and in Thompson's *Laws of*

*Thought* amongst the newer, works that treat of the syllogism. *Same*, though a sign, is scarcely a sign of *quantity*.

The individuality of the middle term and the figure of the syllogism are related. That there are inductions in the first figure has been seen—inductions wherein the correlation between the *ascensus* and *descensus* is at its *maximum* of clearness. Individual terms, however, are better handled as subjects than as predicates, and, for this reason, the Third Figure is generally considered as better adapted to the Inductive Syllogism than the First.

Why, however, does the Third Figure give a universal conclusion when the syllogism is inductive; whereas, when it is other than inductive, it only gives a particular one? The reason of this lies in the details of the conversion. As far as figure is concerned, it makes no difference whether, in such a syllogism as

*All men are mortal;*
*All heroes are men;*
*All heroes are mortal;*

we transpose the first and last propositions *in toto*, or merely transpose the terms of the second. In one case the result is

*Heroes are mortal;*
*Heroes are men;*
*Men are mortal;*

in the other

*Men are mortal;*
*Men are heroes;*
*Heroes are mortal.*

The Figure in each case is the Third. Not so, however, the signs. In the transposition of the *terms*, the transposition which gave us the ordinary syllogism of the third figure, the sign *all* was lost; inasmuch as

*All men are mortal;*

comes out, after conversion,

*Mortals are (some) men.*

In the transposition, however, of the *propositions*, the transposition which gave the Inductive Syllogism with its universal conclusion, the sign *all*, though lost in the subject, was preserved in the predicate.

